# Development of a Contact Glass-Break Detector for the Highest Security Level

**DOI:** 10.3390/s24010097

**Published:** 2023-12-24

**Authors:** Vaclav Mach, Ales Mizera, Pavel Stoklasek, Michaela Karhankova, Milan Adamek, Martin Bednarik

**Affiliations:** 1 Faculty of Applied Informatics, Tomas Bata University in Zlin, Nad Stranemi 4511, 760 05 Zlin, Czech Republic; mizera@utb.cz (A.M.); pstoklasek@utb.cz (P.S.); m_karhankova@utb.cz (M.K.); adamek@utb.cz (M.A.); 2 Faculty of Technology, Tomas Bata University in Zlin, Vavreckova 5669, 760 01 Zlin, Czech Republic; mbednarik@utb.cz

**Keywords:** glass break, detector, accelerometer, alarm system, surveillance

## Abstract

The main object of this research was to develop a security system to evaluate the intrusion into an object through a glass pane. More specifically, this study deals with sensing and evaluating signals from a contact glass-break detector, which is part of an intruder alarm system. Each alarm detector in an alarm system must accomplish certain security level requirements that strictly describe the requirements for the area of use and the detector’s reliability. To date, no contact glass-break detector has been developed and fully tested to meet the stringent requirements of the highest security level. A contact glass-break detector was developed whose main part is an accelerometer that transmits signals from the glass pane. These signals were evaluated according to the developed methodology. It was verified that the proposed system can distinguish at the highest security level between false alarms and situations where the building has been intruded.

## 1. Introduction

Intrusion detection has expanded significantly to meet the growing demand for improved security areas where a person’s life, health, and assets are protected [1,2]. This can be further divided into physical and technical security, where physical security is the most efficient for the general protection of the person. However, physical security is also the most expensive method, and its significant disadvantage is the potential for human error, where even a trained security person can lose focus over the course of several hours [3]. These are the main reasons why technical security is widely used to protect life, health, and assets. Technical security can be described as a collection of security elements, such as safety doors, locks, bars, and cameras, that make breaking into a building or protected areas more difficult, even for experienced intruders [4,5,6]. The most important technical security component is the intruder alarm system (IAS), which can detect the intruder, trigger the alarm, and call for the armed forces to pacify the intruder [7]. The central part of the IAS is the control and indicating equipment, which periodically evaluates the current state of the connected devices, such as detectors [8,9]. The detectors can be divided into several categories according to their specific usage.

The IAS is defined by the EN standard 50131-1:2008 Alarm systems—Intrusion and hold-up systems—Part 1: System requirements [10], where the most important part is focused on the security levels. There are four levels depending on the priority of the security and the capabilities and equipment of the possible intruder. The highest security level (IV) is applied in sectors where security takes priority over all other systems, and intruders can develop a detailed intrusion plan. The intruders also have a full range of equipment and devices, and they can replace critical elements and components in the IAS. This sector includes jewelry stores, art objects in museums, weapons warehouses, the banking sector, and state buildings. The highest security level also states that every part of the security system must be connected to the control and indicating equipment CIE by wires; wireless connections are not acceptable due to the possible jamming of the signal.

The perimeter layer is the most important element between an intruder and the protected assets, usually represented by static components, such as walls, ceilings, and floors, and openable components, such as windows and doors. The aforementioned assets whose protection is desired are usually placed inside a room or building [11,12,13]. Most of the typical perimeter layer is made of bricks, concrete, or wood, where the material is breakable only with a lot of force and noise. The front door is usually the most secure part of the whole technical protection and, thus, not often used for breaking into the building. However, the most fragile and vulnerable parts of the perimeter layer are the glass panes, which an intruder can easily break through. The survey conducted by L. Fennelly et al. [14] stated that more than 80% of intrusions via the perimeter layer take place through a window, which is made of glass panes.

There are two main types of glass-break detectors, namely, acoustic and contact, which are used to detect intrusion through the glass panes. Most of the market offers only acoustic glass-break detectors, which can be placed anywhere in the protected room, where one detector can usually protect several windows at once. This type of glass-break detector uses a piezoelectric microphone to capture the incoming sound pattern typical of breaking glass.

The requirements for contact glass-break detectors are regulated by the European standard EN 50131-2-7-2:2012 Alarm systems—Intrusion and hold-up systems—Part 2-7-2: Intrusion detectors—Glass-break detectors (passive) [15]. The requirements for the glass-break detectors are also divided into four levels based on the assumed knowledge of a potential intruder with respect to the IAS and its technical equipment [16]. However, neither acoustic nor contact detectors that are currently on the market can meet specific requirements for the highest security level given by the standard.

Several publications have attempted to improve acoustic glass-break detectors. For example, K. Lopatka et al. [17] improved the software to be able to distinguish glass breaking from gunshots and screams in noisy environments. Their demonstration proved that the software created for glass-break detectors could decrease the number of generated false alarms. W. Naing et al. [18] designed a new architecture for a glass-break detection approach based on an LSTM deep recurrent neural network to improve the correct detection accuracy with fewer false alarms, which could be used to distinguish the onset of glass breaking from thunder, shouting, or gunshots. Another study conducted by W. Naing et al. [19] introduced a new glass-break detection algorithm design based on a Fuzzy Deep Auto-encoder Neural Network, which can be used in very noisy environments. J. Hart et al. [20] stated that some of the glass-break detectors on the market are not very reliable, especially when a special foil is used. The authors also stated that the contemporary design of detectors can lead to malfunction, which is an essential problem in the case of IASs. M. Lojka et al. [21] created a system for acoustic event detection mainly focused on the detection of gunshots and glass breaking, which could be used to improve the reliability of the glass-break detectors. All of the aforementioned acoustic glass-break detector improvements are nevertheless still incapable of achieving the mentioned highest security level.

A thorough search of prestigious peer-reviewed articles was conducted, and it can be concluded that there has not yet been developed any contact glass-break detection system for glass panes that would meet the highest security level. In our previous studies [22,23], we proposed a new concept of a contact glass-break detector that is physically placed directly on the glass pane and based on an accelerometer, thus being intended to be more sensitive to emerging vibrations. Therefore, the main objective of this research is to develop and verify a contact glass-break detection system that would meet the highest security level requirements according to the European standard EN 50131-2-7-2 [15], as amended.

## 2. Materials and Methods

For the aims of this study, a contact glass-break detector was constructed and tested. In our detector, an accelerometer was used instead of an ordinary shock sensor to precisely measure the energy created by the intrusion into the glass pane. The proposed accelerometer brings more sensitive and accurate measurement results and can also determine the direction of impact. This research used a common accelerometer MPU-6050, which is frequently used in smartphones for measurement of acceleration [24]. The accelerometer is MEMS-based and has an integrated 6-axis motion tracking device with a 3-axis gyroscope, 3-axis accelerometer, and Digital Motion Processor [24]. The raw data from the accelerometer are stored in a 16-bit data register with a range of (0−), which means that the output range is (−32,768; +32,767) for each direction. These raw numbers can be further represented as acceleration by dividing the raw number to obtain the acceleration [25]. However, the final program and the testing part evaluate the raw number in order to reduce the calculation and time of the evaluation by the microcontroller. The output of the MPU-6050 uses 6-bit Analog-to-Digital Converters for digitizing the outputs [26].

**Figure 1:**
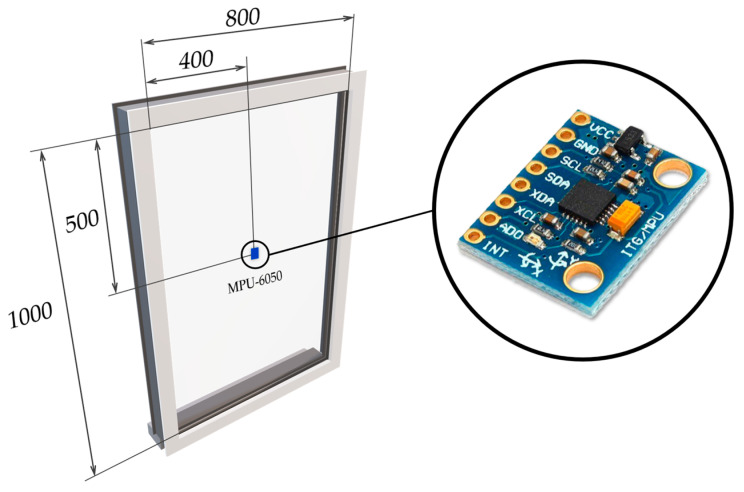
Position of the MPU-6050 on the glass pane (dimensions are in millimeters).

There are several types of glass panes, such as Normal, Coated, Hardened, Insulated, and Wired, in accordance with the said EN standard. However, for purposes of this study, the wired glass pane with dimensions of 800 × 1000 × 6 mm was used due to its usage in the highest security level. The MPU-6050 accelerometer was placed in the center of the glass pane, where the highest deflection is expected during the test. The accelerometer was placed on the inner side of the glass pane, and the impact in the form of the steel ball was performed from the outer side of the glass pane. This ensures that the impact energy is transmitted to the accelerometer in a straight direction. The visible placement of the glass-break detector is intended to deter a potential intruder from breaking the glass pane. Since the detector is used in the highest security level areas, a clear view from the window is not considered important. This placement is recommended by the EN standard; however, the position of the detector on the glass pane is up to the manufacturer. The proposed accelerometer was mounted to the glass pane with a special adhesive. The position of the accelerometer on the glass pane is shown in Figure 1.

**Figure 2:**
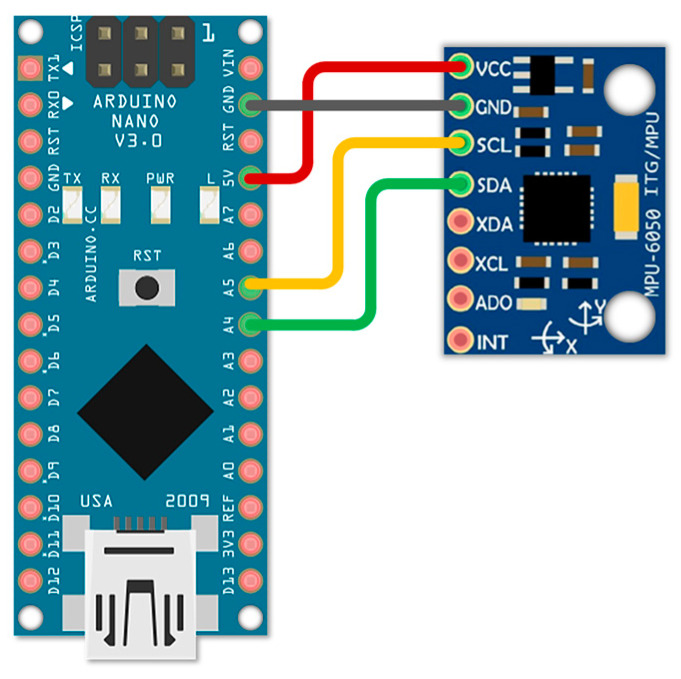
The connection between the MPU-6050 and Arduino Nano.

The main microcontroller connected to the accelerometer MPU-6050 was Arduino Nano, which has the I2C interface needed for communication with the accelerometer. The schematic design can be found in Figure 2. The mentioned microcontroller was connected to the PC, and the incoming data from the accelerometer were processed and analyzed. After the analysis, the final program was created and tested further in this research.

European standard EN 50131-2-7-2:2012 [15] sets multiple testing compliance criteria that must be met for a contact glass-break detector to fulfill the corresponding security level. Testing, according to the standard, is divided into two main groups, namely Performance Tests (PT) and Tests of Immunity to False Alarm Sources (TIFAS). The PT is focused on the events when the alarm must be triggered, whereas the TIFAS is focused on the events when the alarm must not be triggered. This combination ensures the reliability of contact glass-break detectors. This study was focused on glass-break detectors with the highest security level. For the purpose of this study, three tests that apply to detectors of the highest security level were selected from the EN standard. Our testing intended to determine whether the detector we designed can respond to stimuli specified in the EN standard and, in the required cases, trigger an alarm or, on the contrary, prevent a false alarm. The simplified requirements only for the highest security level, which was the main element of this research, are listed in Table 1.

According to Table 1, a glass-break detector must pass both groups of tests, namely the PT, as well as the TIFAS, to be classified for the highest security level. However, it is tough for glass-break detectors to distinguish the PT scenarios from the TIFAS scenarios. The PT scenarios HD and GC must detect the event called the change of the glass integrity. Due to the overlap of the HD or GC vibration level with the IHOHG, the detection of the change in the glass integrity is extremely challenging in the case of contact glass-break detectors. The software of the detector must be able to distinguish between the mentioned scenarios and, thus, must not react to TIFAS while not failing to react to PT.

The first scenario, HD, is focused on the change in the glass pane integrity. This test was performed by drilling a hole with a diameter of 20 mm with a diamond drill bit from the outside of the glass pane. The drilling must be performed in different locations where the first hole was drilled at the furthest point away from the detector on the glass pane and three times at random points on the glass pane.

The second scenario, GC, is also focused on the change in the glass pane integrity where the cutting was performed by a common glass cutter, and the glass pane was carefully scratched in the form of a circle that was 100 mm in diameter. The detector must trigger the alarm after removing the loose part of the glass pane. However, the common glass cutter is not suitable for the cutting of the wired glass pane, which is mostly used at the highest security level. Therefore, a grinder was used instead of a common glass cutter.

**Figure 3:**
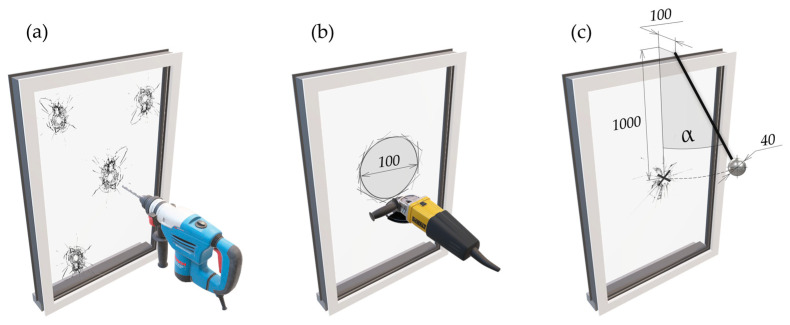
Testing Scenarios: (**a**) hole drilling with a diamond hole saw (HD), (**b**) glass cutting (GC), (**c**) immunity to hard objects hitting the glass (IHOHG).

There are several scenarios in the TIFAS group. However, IHOHG produces the biggest amount of energy, which is the reason why other scenarios of the TIFAS were not used. This scenario focused on the impact of a hard object that was falling to the glass pane in the form of a 40 mm steel ball mounted on a 1 m long pendulum. The ball must be polished, and the impact must be performed without any bouncing. The mentioned scenarios listed in Table 1 are graphically represented in Figure 3.

According to the EN standard, the concept of triggering the alarm has a strict priority. The main priority is the alarm triggering based on the PT and the security level where the alarm must be triggered if the conditions are met. On the other hand, the TIFAS must be recognized, and the detector must not be triggered. The experiment was conducted using the accelerometer MPU-6050 mounted on the glass pane, where the sensor can measure the vibration created by the impact. Measured vibration was further evaluated by the program, and the threshold for the alarm-triggering values was visualized for each scenario. The accelerometer MPU-6050 was chosen for its low cost, availability, and reliability.

## 3. The Main Experiment

The main test used three independent scenarios, which are listed in the mentioned EN standard, namely HD, GC, and IHOHG. The final evaluation of the received data from the accelerometer had to be modified to obtain several threshold levels in a specific time duration. The program can process 512 cycles in one second, and the measured value of the current acceleration is saved in one cycle. The final evaluation of the measured values was performed by converting the negative acceleration value to a positive one and then averaging the values of the last 70 values.

The first scenario is HD, which must trigger the alarm. This scenario created a negligible amount of energy caused by the vibration created by the drilling into the glass pane. The drilling into the glass pane with the drill created much continuous vibration in time, which did not exceed the maximal range of the accelerometer. However, even with the fast-spinning drill, drilling through the glass pane lasted up to a minute. The following scenario GC was very similar to the HD, where the cutting by the grinder also took up to a minute to cut through the glass pane. Both scenarios must trigger the alarm to achieve the highest security level.

**Figure 4:**
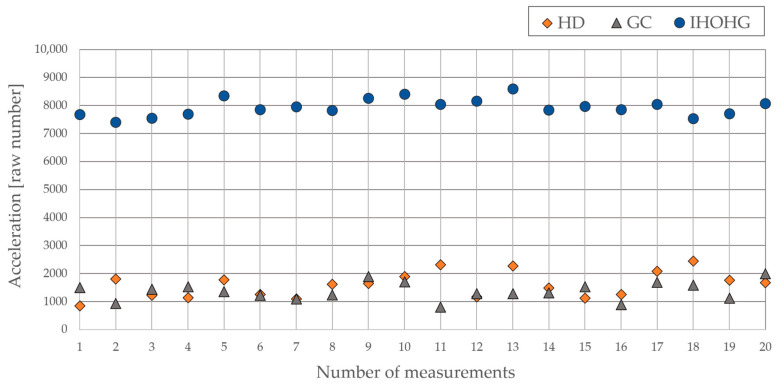
Visualization of the HD, GC, and IHOHG scenarios on the glass pane.

The third scenario, IHOHG, produced the most impact energy, required by the TIFAS. The final program must not trigger the alarm if the acceleration is below the measured visualized values. The measured area below this threshold level also represents the energy on the glass pane without any physical damage. The visualization of all tested scenarios is shown in Figure 4.

The main reliability goal of the detector is to distinguish between the mentioned PT and TIFAS. The detector must not respond to TIFAS scenarios and must respond to the mentioned PT, namely the HD and GC, to be classified for the highest security level.

Based on the measurements and the graphical representation, there is no way to solve the overlapping range using a detector based only on vibrations. At the market, no contact glass-break or acoustic detectors meet the requirements for the highest security level, which is caused by the overlapping of the PT and TIFAS. This problem could be solved by using time analysis based on the duration of each mentioned scenario, where the vibration must be associated with the time duration of the specific scenario.

## 4. Evaluation of the Scenarios

**Figure 5:**
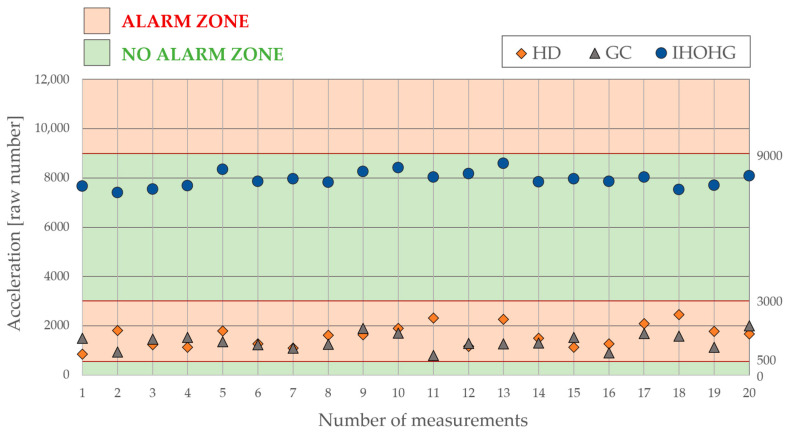
Graphical representation of the threshold values for the final evaluation.

Based on the previous experiment, the specific ranges of acceleration can be assigned to the specific scenarios HD, GC, or IHOHG. This detector has only two ranges or zones: the alarm zone, where the alarm must be triggered, and the no alarm zone, where the alarm must be avoided. A graphical representation of the threshold levels for the final evaluation is shown in Figure 5.

The first range, where the acceleration can cause physical damage to the glass pane, was set at 9000 units up to the maximal limit of the accelerometer, which is 32,767. It means that the values above this threshold must always be evaluated as an alarm. The second range was focused on the IHOHG scenario, which always produced less acceleration than 9000 units, down to zero. The acceleration in this range must not be classified as an alarm. However, in the mentioned range from 9000 to 0, there must be another range for the last two scenarios, HD and GC, which overlap with the IHOHG. This range follows the values measured in the main experiment, where the range for both scenarios was set from 500 up to 3000 units. However, this time, the range depends on the time duration of the event. The cutting or drilling can take up to one minute, which increases the average value of the acceleration. On the other hand, the IHOHG takes less than one second. That is the reason why the scenarios HD and GC are dependent on time. The last range from 500 down to 0 units must be classified as a non-alarm condition. The idle acceleration of the glass pane is less than 500. The following Figure 5 shows the graphical representation of the individual threshold values for the final program.

**Figure 6:**
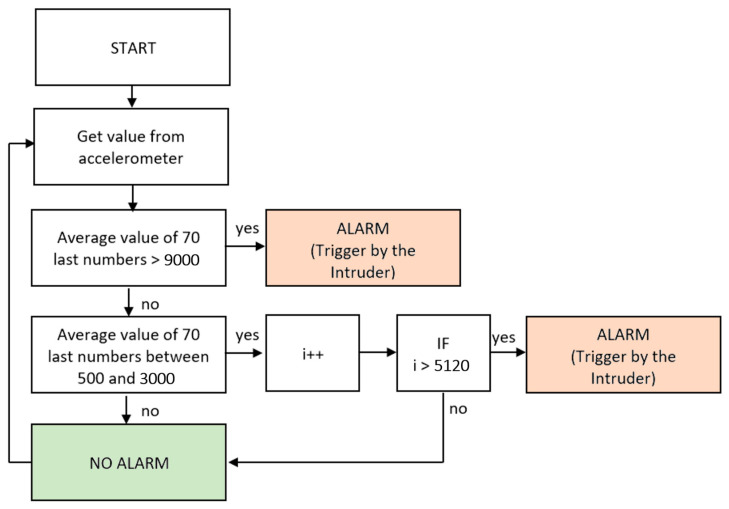
Flowchart of the final program.

The final program of the glass-break detector must properly evaluate the mentioned threshold values from Figure 5. The flowchart of the final program is listed in the following Figure 6.

At the beginning of the program, the current value of the accelerometer was read from the device by the I2C interface, and it was stored in the program memory. The program automatically stores the last 70 values and calculates the average value of these values.

The first condition was based on the value 9000, determined by the experiment. When the average value of the last 70 values exceeds the 9000 level, the alarm is triggered. The produced energy over this acceleration limit can break the glass pane.

The second condition was focused on the overlapping of the mentioned HD, GC, and IHOHG, where the time duration of the scenario is essential. Scenarios HD and GC create an average value of acceleration between 500 and 3000, but the program needs to distinguish between the mentioned PT and TIFAS. That is the reason for the specific time duration requirement, where the alarm is triggered only if the vibration in the 500 and 3000 range lasts a specific duration time. The program needs to have a counter for the mentioned range. The threshold time for the counter of HD and GC is set to 10 s. The alarm must be triggered if the vibration in the 500 and 3000 range lasts longer than 10 s. One cycle of the program lasts 1.953 ms, which means that 10 s cover exactly 5120 program cycles. This time duration was used only for the purpose of the measurement to prove the concept. The real-time duration of the final prototype must be much shorter. The maximum intended time of the alarm triggering should be 1 s. This short time avoids resetting the timing by hitting the glass with a heavy object while drilling it.

## 5. Verification of the Final Program

**Figure 7:**
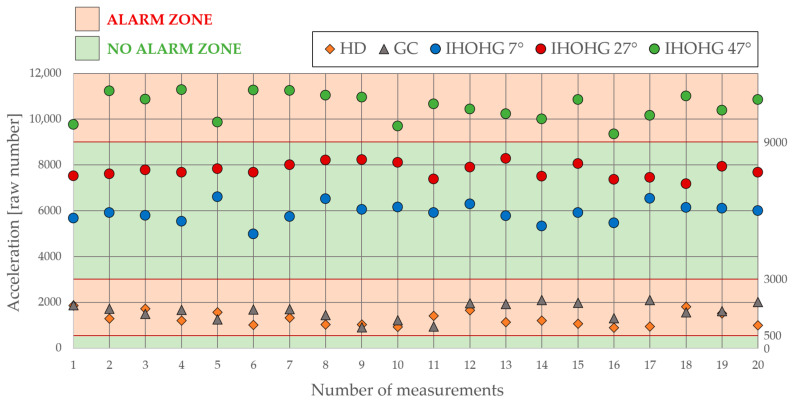
Graphical representation of the verification experiment.

Based on the previous threshold values and flow diagram, the final program was created. The reliability of the program must be verified by the experiment mentioned above with different input parameters. The verification has to be carried out for all three scenarios. The first is HD, which has no adjustable ways of performing. The same applies to GC, where both scenarios are performed exactly as in the experimental part. However, the third scenario, IHOHG, can be modified to test the threshold level. This modification can be achieved by increasing and decreasing the pendulum’s angle, where the steel ball will gain a different amount of energy. The outcome of the verification experiment can be found in the following Figure 7.

A new series of measurements was performed for all tested scenarios to test the reliability of the program. The HD and GC scenarios had the same setting. However, the IHOHG was performed three times for different angles of the pendulum. A standard 27° angle was used to verify the experiment. However, to test the reliability of the created program even more, the experiment used 7° and 47° angles of the pendulum, where at 7° the alarm must not be triggered, and at 47° the alarm must be triggered. The final testing results are shown in Figure 7. It was verified that the proposed system could be used in an environment with the highest security level, where an increased risk of object intrusion is expected.

## 6. Results and Discussion

The main emphasis of this article was the possibility of creating a program for a contact glass-break detector to meet the highest criteria given by the current EN standard, namely the highest security level. The testing according to the EN standard for PT and TIFAS was carried out using the accelerometer as the main measuring device. Three combined scenarios were described, created, and measured according to the EN standard with the graphical representation shown in Figure 7.

The main experiment established vibration threshold levels 500, 3000, and 9000, which divided the whole range of the accelerometer into four sections. Namely above 9000 as an alarm, 9000–3000 as no alarm, 3000–500 with time duration as an alarm, and 500–0 as no alarm. The designed algorithm for a glass-break detector with the mentioned threshold levels can be used in sectors with the highest security level, according to the EN standard. These sectors include jewelry stores, art objects in museums, weapons warehouses, the banking sector, and state buildings.

A new detector must be constructed to create a fully functional prototype of the proposed concept of a glass-break detector with the proposed algorithm. This new model must be connected to the CIE by wires to achieve the highest security level. Only the finished prototype of the glass-break detector can be tested for electromagnetic compatibility and sabotage, which are also mentioned by the EN standard. A sabotage in the form of dismounting the sensor from the glass pane or electromagnetic impulse is handled by the CIE through periodic requests of the state. When the sensor is dismounted, destroyed, jammed, or disconnected, the CIE automatically triggers the alarm.

Furthermore, some events do not create any vibrations and cause a change in the glass’s integrity, e.g., flame cutting or acid dissolution of the glass pane. Other scenarios are created by some external events, like hailstorms or storms, which are not considered by the EN standard. However, these unpredictable weather or climate effects can also damage the integrity of a glass pane or just trigger a false alarm that must be eliminated by the system. It is impossible to deactivate the individual parts of a security system that permanently report the state of building disturbances due to adverse weather conditions. However, this condition can occur, but the building must still be protected from a potential intruder.

## 7. Conclusions

The article focused on the development of a system for securing glass panes against mechanical damage. The proposed system consists of a commonly used MPU-6050 accelerometer, which stands out for its simplicity and functionality, which is very important for securing buildings with the highest security level. Any damage or malfunction of the detector is inadmissible in this security area. Furthermore, the proposed system consists of an Arduino development platform, which ensures the transfer and evaluation of data according to a specially designed algorithm. It has been proven that the developed system for detecting the destruction of glass panes is fully functional and can be used in systems at the highest security level. To verify its functionality, three scenarios (HD, GC, and IHOHG) were used, which the proposed system complied with. Based on the measured and evaluated data, it can be concluded that this simple but fully functional system for detecting the destruction of glass panes can be used in applications for the highest security level.

According to selected passages from the EN 50131-2-7-2 standard [15], this system can be used for buildings with the highest security level; however, in this standard, there is no mention of disruption by adverse weather conditions, such as storms, wind, and hail. Therefore, it is essential to further research and verify whether the proposed system of intrusion detection through a glass pane is fully functional even in the event of adverse weather effects, which may enter the data evaluation process and disrupt the now fully functional evaluation algorithm. The system detects and continuously evaluates vibrations or impacts to a glass pane and, based on a sophisticated algorithm, evaluates whether it is a violation of the glass pane or whether there is only a permissible vibration of the windowpane that does not present any danger of breaking through. This proposed system of detecting a glass pane intrusion is a mere part of the whole security system, which must be fully functional, and these sub-systems must complement each other to fully ensure the protection of the entire building at the highest security level.

## Figures and Tables

**Table 1:** Requirements for the highest security level of a contact glass-break detector.

Testing Scenarios	Risk Level IV
Hole Drilling with a diamond hole saw (HD)	Alarm must be triggered
Glass Cutting (GC)	Alarm must be triggered
Immunity to Hard Objects Hitting the Glass (IHOHG)	Alarm must be avoided

## Data Availability

Data will be made available after reasonable request.
